# Inhibition of 11β-HSD1 by Tetracyclic Triterpenoids from *Euphorbia kansui*

**DOI:** 10.3390/molecules171011826

**Published:** 2012-10-09

**Authors:** Jie Guo, Li-Yan Zhou, Hong-Ping He, Ying Leng, Zhen Yang, Xiao-Jiang Hao

**Affiliations:** 1 Laboratory of Chemical Genomics, School of Chemical Biology and Biotechnology, Peking University Shenzhen Graduate School, Shenzhen 518055, Guangdong, China; Email: guojie@pkusz.edu.cn (J.G.); zhouly@pkusz.edu.cn (L.-Y.Z.); zyang@pku.edu.cn (Z.Y.); 2 Key Laboratory of Bioorganic Chemistry and Molecular Engineering, Ministry of Education and Beijing National Laboratory for Molecular Science, College of Chemistry, Peking University, Beijing 100871, China; 3 State Key Laboratory of Phytochemistry and Plant Resources in West China, Kunming Institute of Botany, Chinese Academy of Sciences, Kunming 650204, Yunnan, China; Email: hehongping@mail.kib.ac.cn; 4 Shanghai Institute of Materia Medica, Chinese Academy of Sciences, Shanghai 201203, China; Email: yleng@mail.shcnc.ac.cn

**Keywords:** tetracyclic triterpenoids, *Euphorbia kansui*, inhibition of 11β-HSD1, docking

## Abstract

The roots of *Euphorbia kansui* are considered an important traditional folk medicine. In this study the ethanol extracts of *E. kansui* were investigated. A new tetracyclic triterpenoid, euphane-3β,20-dihydroxy-24-ene, in addition to five known triterpenoids with euphane skeletons were isolated. Their structures were elucidated on the basis of physical and spectral techniques (1D-, 2D-NMR and MS, respectively). Furthermore, these compounds **1**–**6** exhibited strong inhibitory activity against human 11β-hydroxysteroid dehydrogenase type 1 (11β-HSD1), with IC_50_ values of 34.86 nM, 1.115 μM, 16.08 nM, 2.815 nM, 26.47 nM, 15.99 nM, and 41.86 nM, respectively. The docking results show that the ring part of compounds can insert into the hydrophobic core of h11β-HSD1 and the alkane chain orientates toward the outside. The results presented herein provide a scientific explanation for the usage of the *E. kansui* in clinical treatment of diabetes.

## 1. Introduction

Type 2 diabetes mellitus is a complex endocrine and metabolic disorder. The interaction between several genetic and environmental factors results in a heterogeneous and progressive disorder with variable degrees of insulin resistance and pancreatic β-cell dysfunction [1]. In China it is proving to be a major public health problem, especially in the urban areas. The increasing prevalence, variable pathogenesis natural history, and complications of type 2 diabetes emphasize the urgent need for new treatment strategies [2]. Herbal supplements for diabetes should be a part of a holistic approach to treatment that addresses proper nutrition, a good exercise program, and continued monitoring of blood glucose levels. 

Plants of the *Euphorbia* genus produce structurally unique and diversified diterpenoids and triterpenoids, which have attracted great interest from the biogenetic, synthetic, biological and toxicological points of view [3,4,5,6,7]. Certain types of triterpenoids isolated from plants of the genus *Euphorbia*, such as euphanes and tirucallanes, may be the ultimate biogenetic precursors of limonoids which have recently attracted attention because compounds belonging to this group have exhibited a range of biological activities like insecticidal, insect antifeedant and growth regulating activity on insects as well as antibacterial, antifungal, antimalarial, anticancer, antiviral and a number of other pharmacological activities in humans [8]. Therefore, the triterpenoids and its derivatives produced by genus *Euphorbia* have a lot of future in studies of chemical components and biological activities.

**Figure 1:**
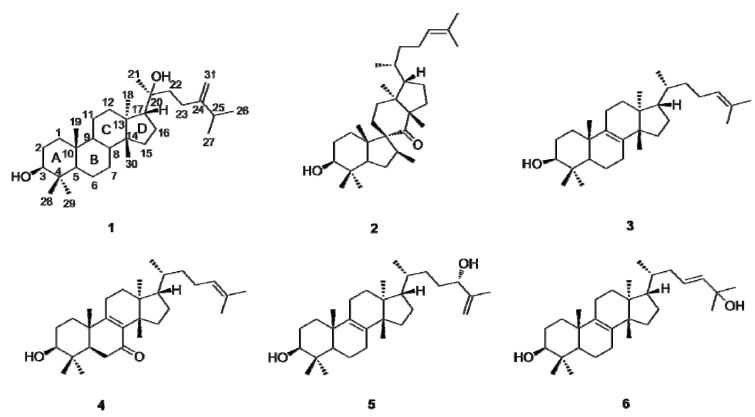
Structures of the triterpenoids **1**–**6** isolated from *Euphorbia kansui*.

*Euphorbia kansui* Lour. (Euphorbiaceae) is a vivacious herb distributed in the central and western parts of China. The dried roots of *Euphorbia kansui* have been used as an herbal remedy for edema, ascites, and cancer in mainland China [9]. Previous phytochemical investigations on this species yielded a number of ingenol diterpenoid esters and jatrophane diterpenoids [10,11]. In the course of our search for bioactive natural products from the roots of *E. kansui*, six tetracyclic triterpenoids **1**–**6** (Figure 1) were isolated from the EtOH extracts of this plant. Herein we describe the isolation and characterization of the new triterpenoid, along with five known triterpenoids, and the evaluation of inhibitory activity against human and mouse 11β-HSD1.

## 2. Results and Discussion

### 2.1. Identification of Compounds

Compound **1** was obtained as a colorless powder. Its molecular formula was determined to be C_31_H_54_O_2_ by HR-TOF-MS (*m/z* [M+Na]^+^ 481.4173, calcd. 481.4124). The IR absorption bands indicated the presence of double bond (1639 cm^–1^) and hydroxyl (3439 cm^–1^) groups. The 1D NMR spectra exhibited resonances for six quaternary, eleven methylene, six methine, and eight methyl carbons, which were assigned to a terminal double bond, one oxygenated quaternary, one oxygenated methine, two secondary methyls, six tertiary methyls, and two hydroxyl groups. As the molecular formula indicated the presence of five units of unsaturation, the compound must therefore be tetracarbocyclic since there is only one terminal double group. 

Comparison of the ^1^H- and ^13^C-NMR data of **1** with those of euphorbol [12], a tirucallane-type triterpenoid isolated from the same plant, revealed that both compounds were characterized by similar chemical shifts, suggesting a common structural motif they shared, except for the signals due to the olefinic group and the side chain part. Two hydroxyls, as required by its molecular formula and IR spectra, were located at positions C-3 and C-20 based on their chemical shifts and HMBC correlations from H-3 to Me-28,29, and Me-21 to C-20. The detailed analysis of **1** using ^1^H-^1^H COSY and HMQC techniques disclosed three partial structural units, between H-3 and H-2; between H-2 and H-1; between H-5 and H-6; between H-6 and H-7; between H-7 and H-8; between H-8 and H-9; between H-9 and H-11; between H-11 and H-12, and between H-15 and H-16, and between H-16 and H-17. This was also supported by analysis of the HMBC spectrum, which showed two- and three-bond correlations between H-1 and C-2; between H-2 and C-3; between H-5 and C-6; between H-6 and C-7; between H-7 and C-8; between H-8 and C-9; between H-9 and C-11; between H-11 and C-12; between H-15 and C-17; and between H-16 and C-17.

**Figure 2:**
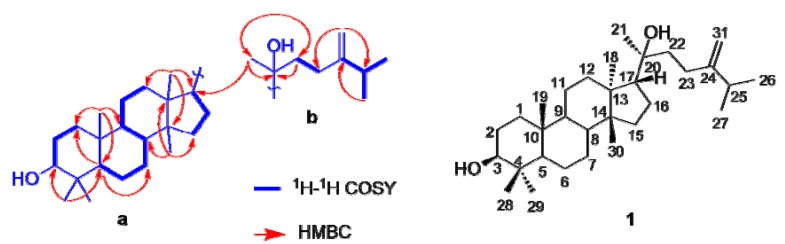
Selected two-dimensional NMR correlations of compound **1**.

Furthermore, the HMBC correlations of the five individual tertiary methyl signals on rings A–D [between Me-28 (*δ*_H_ 0.96) and C-29, C-4, C-3, and C-5; between Me-29 (*δ*_H_ 0.77) and C-28, C-4, C-3, and C-5; between Me-19 (*δ*_H_ 0.84) and C-1, C-5, C-9, and C-10; between Me-30 (*δ*_H_ 0.88) and C-8, C-13, C-14, and C-15; between Me-18 (*δ*_H_ 0.96) and C-12, C-13, C-14, and C-17] firmly established the linkages of these partial structural units (substructure a). The side chain (substructure b) was assembled by ^1^H-^1^H COSY correlations between H-25/Me-26,27 and H-22/H-23 as well as HMBC correlations of H-31/C-25, H-31/C-23, Me-26,27/C-24, Me-21/C-22, H-22/C-20 and Me-21/C-20. The two substructures should join at C-17 confirmed by the HMBC cross peaks between Me-21 and C-17 and between H-16 and C-20. Therefore, a planar structure of **1** was derived, as shown in Figure 2.

**Figure 3:**
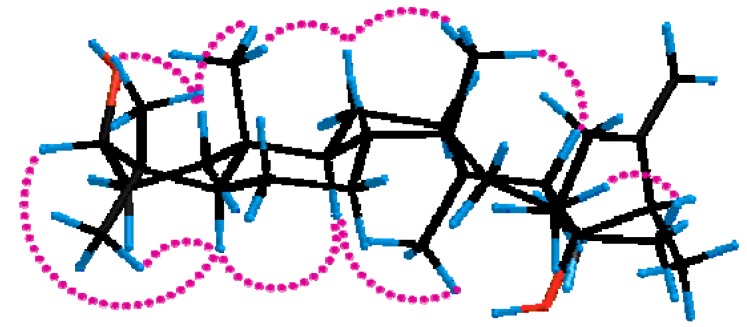
Key ROESY correlations of compound **1**.

The relative configuration of **1** was determined by ROESY experiments (Figure 3). The large coupling constant (*J*_2,3_ = 10.8 Hz) of H-3 indicated that the hydroxyl group was oriented equatorially (*β*) at C-3 [13]. The relative configurations of the methyl groups and other protons in the rings A–D were ascertained on the basis of the ROESY correlations. The significant ROESY correlations of H-3/H-5, H-3/Me-28, H-5/H-9, and Me-18/H-9 indicated that H-3, H-5, Me-28, H-9, and Me-18 were cofacial, adopting an *α*-orientation. The ROESY cross-peaks of Me-19/H-1*β*, Me-19/H-12β, Me-30/H-12β, and Me-30/H-17 indicated that Me-19, Me-30 and H-17 was *β-*oriented. Furthermore, ROESY correlations between Me-21 and CH_2_-16 and the absence of NOE between Me-18 and Me-21 were consistent with those of euphane-type triterpenoids [13,14]. And the positive optical rotation of **1** (+13.4°) also indicated that **1** belonged to the euphane rather than the tirucallane series [15,16,17]. Thus, compound **3** was elucidated to be euphane-3β,20-dihydroxy-24-ene.

### 2.2. Inhibition of 11β-HSD1

The oxidoreductase 11β-hydroxysteroids dehydrogenase type 1 (11β-HSD1) mainly catalyzes the intracellular regeneration of active GCs (cortisol, corticosterone) from inert inactive 11-keto forms (cortisone) in liver, adipose tissue and brain, amplifying local GC action. Multiple lines of evidence have indicated that 11β-HSD1-mediated intracellular cortisal production may have a pathogenic role in type 2 diabetes and its co-morbidities. 11β-HSD1 becomes a novel target for anti-type 2 diabetes drug development, and inhibition of 11β-HSD1 offers a potential therapy to attenuate the type 2diabetes [18]. The inhibitory effects of compounds **1**−**6** on mouse and human 11β-HSD1 and 11β-HSD2 were evaluated (Table 1). All assays were carried out in duplicate with glycyrrhizinic acid and carbenoxolone as positive controls. All the tested compounds **1**–**6** have a significant inhibition of both mouse and human 11β-HSD1, among them compound **4** shows the strongest inhibitory effect on mouse and human 11β-HSD1 inhibition with IC_50_ of 13.36 and 2.815 nM, respectively. Compounds **2**–**5** have IC_50_>1 mM against mouse 11β-HSD2; however, only compound **1** has a good selectivity against human 11β-HSD2, the IC_50_ was 8.179 μM and selectivity between HSD2/HSD1 was 234.6 times, the remaining compounds HSD2/HSD1 selectivity are less than 100 times. In Traditional Chinese Medicine, processed *E. kansui* has been used as a herbal remedy for diabetes [19]. The results presented herein provide a scientific explanation for the usage of the *E. kansui* in clinical treatment. 

**Table 1:** Inhibition of 11β-HSD1.

Compounds	Mouse 11β-HSD1 (IC_50_)	Mouse 11β-HSD2 (IC_50_)	Mouse HSD2/HSD1	Human 11β-HSD1 (IC_50_)	Human 11β-HSD2 (IC_50_)	Human HSD2/HSD1
Compound **1**	78.44 nM	>1 mM	>12748	34.86 nM	8.179 μM	234.6
Compound **2**	1.077 μM	>1 mM	>928	1.115 μM	2.626 μM	2.35
Compound **3**	80.52 nM	>1 mM	>12419	16.08 nM	0.3952 μM	24.6
Compound **4**	13.36 nM	>1 mM	>74850	2.815 nM	0.107 μM	38
Compound **5**	49.46 nM	>1 mM	>20218	26.47 nM	1.687 μM	63.7
Compound **6**	294.7 nM	>1 mM	>3393	15.99 nM	0.6664 μM	41.7

### 2.3. In Silico Study of the Activities of Compounds

**Figure 4:**
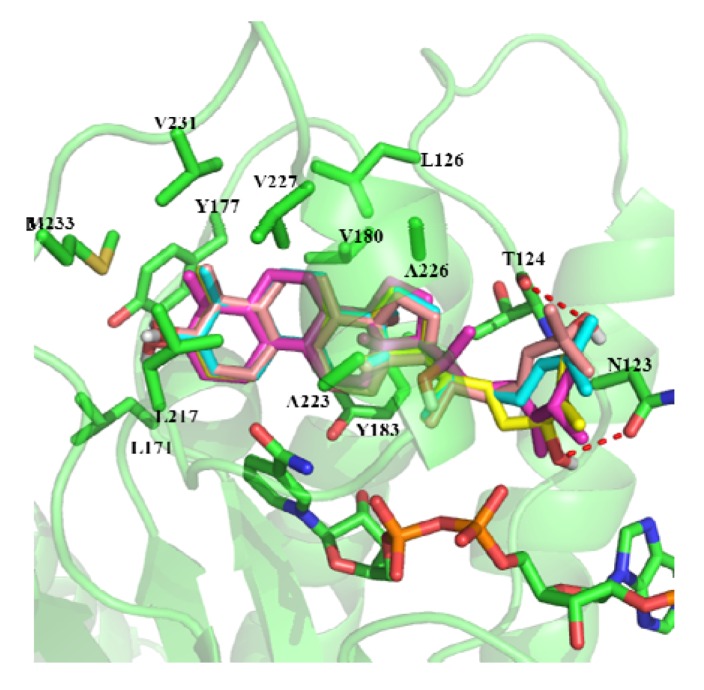
The binding mode of compounds **1**,**3**,**5**, and **6**. Purple: compound **1**; Cyan: compound **3**; Yellow: compound **5**; Orange: compound **6**.

In order to investigate the activity difference against h11β-HSD1 of these compounds, we predicted their binding mode using molecular docking. The docking results show that the binding modes of compound **1**, **3**, **5** and **6** are similar (Figure 4). The ring part of compounds inserts into a hydrophobic core composed of Leu126, Leu171, Tyr177, Val180, Tyr183, Leu217, Ala223, Ala226, Val227, Val231, Met233 and the cofactor NADP. The alkane chain orientates towards the outside. The C-24 hydroxyl of compound **5** can form hydrogen bonds with the side chain of Asn123. The C-21 hydroxyl of compound **6** can form hydrogen bonds with the backbone carbonyl group of Thr124.

**Figure 5:**
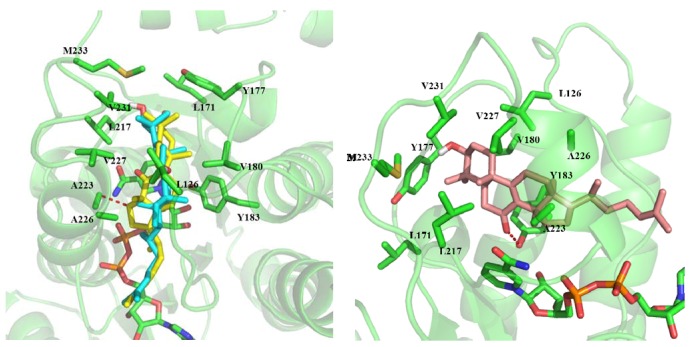
The binding mode of compound **2** and **4**. Cyan: compound **3**; Orange: compound **2**; Yellow: compound **4**.

Compound **2** is a rearranged euphane triterpenoid containing a contracted five-membered ring B. Although the ring part of compound **2** can also insert into the hydrophobic core, the C-15 position has a steric clash with the residue Ala223. The ring part of compound **4** also can insert into the core composed of hydrophobic residues. The C-7 carbonyl of compound **4** can form hydrogen bonds with the side chain of Tyr183. Kelly *et al*. [20] have shown that hydrogen bonds can be stronger by up to 1.2 kcal/mol when they are sequestered in hydrophobic surroundings than when they are solvent exposed. This may be the reason that the activity of compound **4** is a little higher than that of the other compounds (Figure 5). 

**Figure 6:**
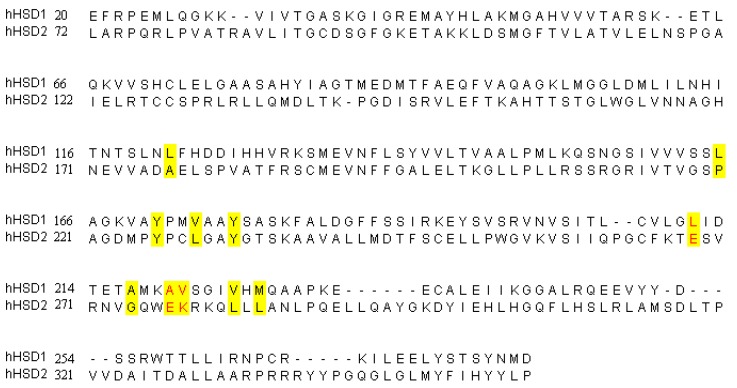
The sequence alignments of hHSD1 and hHSD2, the residues formed binding pocket were colored yellow.

To further investigate the mechanism(s) of compound selectivity between h11β-HSD1 and h11β-HSD2, the residues lining the binding pocket of h11β-HSD1 and h11β-HSD2 were compared (Figure 6). It can be found that most of the binding pocket residues are similar except three residues (colored red). The residues constituted the binding pocket in h11β-HSD1 are hydrophobic. As compared with h11β-HSD1, three residues constituted the binding pocket in h11β-HSD2 are hydrophilic (Glu217, Glu226 and Lys227, colored red). These hydrophilic residues may affect that the ring part of compounds inserts into the binding pocket of h11β-HSD2. This may account for the high h11β-HSD1 selectivity of these compounds. 

## 3. Experimental

### 3.1. General

Optical rotation was measured on a Perkin-Elmer model 241 polarimeter. The IR spectrum was measured in a Bio-Rad FTS-135 spectrometer with KBr pellets. FAB, EI and high-resolution mass spectra were recorded using a Finnigan MAT 90 instrument and VG Autospec-3000 spectrometer respectively. ^1^H- and ^13^C-NMR spectra were measured on a Bruker AM-400 spectrometer, while 2D NMR spectra were recorded on a Bruker DRX-500 instrument. Chemical shifts were reported using residual CDCl_3_ (*δ*_H_ 7.26 and *δ*_C_ 77.0) as internal standard. Column chromatography was performed on silica gel H (10–40 μm; Qingdao Marine Chemical Inc., Qiangdao, China), C18 silica gel (20–45 μm; Chromatorex, Tokyo, Japan), Precoated silica gel GF254 and HF254 plates (Qingdao Haiyang Chemical Plant, Qingdao, China) were used for TLC.

### 3.2. Plant Material

The roots of *Euphorbia kansui* Lour. were collected in Kuitun of Gansu Province, China, in December 2007, and identified by Xun Gong, Kunming Institute of Botany, Chinese Academy Sciences, Kunming, Yunnan, China.

### 3.3. Extraction and Isolation

The dried and powdered roots of *E. kansui* (20 kg) were extracted with 95% EtOH (40 L, 3 times, 60 °C). Removal of the solvent gave a crude residue (760 g), which was partitioned between petroleum ether (280 g), EtOAc (56 g), and H_2_O (420 g). The petroleum ether extracts was applied to a silica gel column (200–300 mesh), eluting with gradient mixtures of petroleum ether-acetone (from 1:0 to 0:1) to give seven major fractions (Fr1–Fr7). Fr1 (80 g) was chromatographed on a silica gel (200–300 mesh) column eluted with petroleum-ether/EtOAc, (50:1) to afford **3** (979 mg). Fr5 (50 g) was subjected to amino silica gel and silica gel CC (300–400 mesh), eluting with petroleum ether/EtOAc (from 10:1 to 3:1) to afford four major subfractions, Fr5a–Fr5d. Fr5b (2.7 g) was further purified by silica gel CC (silica gel H, petroleum ether/acetone, 18:1) and Sephadex LH-20 column (petroleum ether/CHCl_3_/MeOH, 2:1:1) to obtain **2** (18 mg) and **1** (42 mg). Fr6 (39 g) was applied to a MPLC, eluted with CH_3_OH/H_2_O (3:5 to 1:0) to afford three major subfractions, Fr6a–Fr6c. Fr6a (2.2 g) was further purified by silica gel CC (silica gel H, petroleum ether/EtOAc, 20:1) and Sephadex LH-20 column (petroleum ether/CHCl_3_/MeOH, 2:1:1) to obtain **4** (20 mg). The EtOAc (56 g) phase was subjected to column chromatogratography on RP-18 silica gel eluted with MeOH/H_2_O (6:4 to pure MeOH), to furnish five fractions (EFr1–EFr5). The fourth fraction (MeOH/H_2_O 9:1, 3 g) was rechromatographed on a Sephadex LH-20 column (MeOH) and further purified by silica gel CC (Silica gel H, petroleum ether/acetone, 12:1) to yield compounds **5** (12 mg) and **6** (5 mg).

*Euphane-3β,20-dihydroxy-24-ene* (**1**), Formula: C_31_H_54_O_2_; colorless oil; [α]_D_^20^ +13.4° (c 0.30, MeOH); IR ν_max_ (KBr) 3439, 3086, 2958, 2871, 1044 cm^−1^; EI-MS *m/z* 458 [M]^+^; HR-TOF-MS *m/z* 481.4173. ^1^H-NMR: 1.70 (1H, m, 1α-H), 0.96 (1H, m, 1β-H), 1.62 (2H, m, 2-CH_2_), 3.21 (1H, dd, *J* = 4.8, 10.8 Hz, 3-H), 0.72 (1H, d, *J* = 11.6 Hz, 5-H), 1.46 (2H, m, 6-CH_2_), 1.74 (1H, m, 7α-H), 1.33 (1H, m, 7β-H), 1.72 (1H, m, 8-H), 1.31 (1H, m, 9-H), 1.50 (1H, m, 11α-H), 1.26 (H, m, 11β-H), 1.26 (1H, m, 12α-H), 1.57 (1H, m, 12β-H), 1.47 (1H, m, 15α-H), 1.07 (1H, m, 15β-H), 1.88 (1H, m, 16α-H), 1.26 (1H, m, 16β-H), 1.75 (1H, m, 17-H), 0.96 (3H, s, 18- Me), 0.84 (3H, s, 19- Me), 1.13 (3H, s, 21- Me), 1.57 (1H, m, 22-CH_2_), 2.10 (2H, m, 23-CH_2_), 2.25 (1H, septet, 25-H), 1.06 (6H, d, *J* = 6.0 Hz, 26- Me and 27- Me), 0.97 (3H, s, 28- Me), 0.77 (3H, s, 29- Me), 0.88 (3H, s, 30- Me), 4.74 and 4.68 (2H, s, 31-CH_2_). ^13^C-NMR: 39.0 (C-1), 27.3 (C-2), 78.9 (C-3), 38.9 (C-4), 55.8 (C-5), 18.4 (C-6), 25.3 (C-7), 42.1 (C-8), 50.4 (C-9), 37.2 (C-10), 21.4 (C-11), 32.5 (C-12), 49.9 (C-13), 40.3 (C-14), 31.0 (C-15), 27.5 (C-16), 49.5 (C-17), 16.5 (C-18), 15.6 (C-19), 75.7 (C-20), 23.7 (C-21), 40.5 (C-22), 28.0 (C-23), 156.4 (C-24), 34.1 (C-25), 21.9 (C-26), 21.9 (C-27), 28.0 (C-28), 15.4 (C-29), 16.1 (C-30), 106.1 (C-31).

*Kansuinone* (**2**), Formula: C_30_H_50_O_3_; colorless oil; [α]_D_^16^ +12.4° (c 0.19, MeOH); UV (MeOH) λmax 202.2 nm; IR (KBr) ν_max_: 3432, 2963, 2928, 1675, 1629, 1460, 1378, 1022 and 584 cm^−1^; EI-MS *m/z* 458 [M]^+^; the ^1^H-NMR and ^13^C-NMR data in accordance with the literature [21]. ^1^H NMR: 2.10 (1H, m, 1α-H), 1.20 (1H, m, 1β-H), 1.67 (2H, m, 2-H), 3.49 (1H, dd, *J* = 6.4, 9.2 Hz, 3-H), 2.68 (1H, dd, *J* = 14.4, 6.0 Hz, 5-H), 2.16 (1H, m, 6α-H), 1.43 (1H, m, 6β-H), 4.26 (1H, t, *J* = 7.2 Hz, 7-H), 2.13 (1H, m, 11α-H), 1.64 (1H, m, 11β-H), 1.88 (1H, m, 12α-H), 1.79 (1H, m, 12β-H), 1.76 (1H, m, 15α-H), 1.30 (1H, m, 15β-H), 1.31 (1H, m, 16α-H), 1.88 (1H, m, 16β-H), 1.62 (1H, m, 17-H), 0.69 (3H, s, 18-H), 0.90 (3H, s, 19-H), 1.46 (1H, m, 20-H), 0.86 (3H, d, *J* = 6.4 Hz, 21-H), 1.55 (1H, m, 22α-H), 1.13 (1H, m, 22β-H), 1.88 (1H, m, 23α-H), 2.00 (1H, m, 23β-H), 5.07 (1H, dd, *J* = 7.0, 1.2 Hz, 24-H), 1.68 (3H, s, 26-H), 1.60 (3H, s, 27-H), 0.99 (3H, s, 28-H), 0.88 (3H, s, 29-H), 1.16 (3H, s, 30-H). ^13^C-NMR: 29.7 (C-1), 28.2 (C-2), 79.3 (C-3), 37.8 (C-4), 47.8 (C-5), 34.7 (C-6), 76.4 (C-7), 219.3 (C-8), 61.6 (C-9), 49.0 (C-10), 24.2 (C-11), 31.5 (C-12), 45.9 (C-13), 61.8 (C-14), 29.7 (C-15), 26.8 (C-16), 49.6 (C-17), 16.8 (C-18), 17.6 (C-19), 35.2 (C-20), 18.6 (C-21), 35.3 (C-22), 24.6 (C-23), 124.8 (C-24), 131.5 (C-25), 25.7 (C-26), 17.7 (C-27), 29.7 (C-28), 16.6 (C-29), 22.4 (C-30).

*Euphol* (**3**), Formula: C_30_H_50_O; colorless needle; m.p. 165–167 °C; [α]_D_^20^ +32.0° (c 0.30, MeOH); ESI-MS *m/z*: 427 [M+H]^+^, 411, 393, 109, 69; the ^1^H-NMR and ^13^C-NMR data in accordance with the literature [22]. ^1^H-NMR: 0.75 (3H, s, 18-H), 0.79 (3H, s, 29-H), 0.84 (3H, s, 30-H), 0.86 (3H, d, *J* = 6.4 Hz, 2l-H), 0.95 (3H, s, 28-H), 1.00 (3H, s, 19-H), 1.60 (3H, s, 26-H),1.66 (3H, s, 27-H), 3.21 (1H, dd, *J* = 11.5, 4.5 Hz, 3-H), 5.08 (1H, t, *J* = 7.0 Hz, 24-H). ^13^C-NMR: 35.73 (C-1), 24.28 (C-2), 79.06 (C-3), 38.96 (C-4), 50.61 (C-5), 18.35 (C-6), 27.89 (C-7), 134.58 (C-8), l34.58 (C 9), 36.29 (C-10), 21.09 (C-11), 28.22 (C-12), 44.65 (C-13), 49.93 (C-14), 30.93 (C-15), 31.16 (C-16), 50.56 (C-17), 15.82 (C-18), l9.16 (C-19), 36.44 (C-20), 18.69 (C-21), 35.64 (C-22), 25.01 (C-23), 125.34 (C-24), l31.00 (C-25), 17.59 (C-26), 25.64 (C-27), 26.61 (C-28), 28.0l (C-29),15.40 (C-30).

*Kansenone* (**4**), Formula: C_30_H_48_O_2_; colorless oil; [α]_D_^20^ +14.1° (c 0.4, MeOH); ESI-MS *m/z*: 463 [M+Na]^+^, 425, 407, 327, 273, 69; the ^1^H-NMR and ^13^C-NMR data in accordance with the literature [23]. ^1^H-NMR: 1.45 (1H, m, 1α-H), 1.86 (1H, dd, *J* = 13.1, 3.3 Hz, 1β-H), 1.75 (1H, m, 2α-H), 1.67 (1H, m, 2β-H), 3.29 (1H, dd, *J* = 4.6, 11.6 Hz, 3-H), 1.67 (1H, m, 5-H), 2.41 (1H, dd, *J* = 3.9, 15.8 Hz, 6α-H), 2.38 (1H, dd, *J* = 12.4, 15.8 Hz, 6β-H), 2.37 (1H, m, 11α-H), 2.24 (1H, m, 11β-H), 1.76 (1H, m, 12α-H), 1.80 (1H, m, 12β-H), 1.56 (1H, m, 15α-H), 2.13 (1H, m, 15β-H), 1.33 (1H, m, 16α-H), 1.93 (1H, m, 16β-H), 1.43 (1H, m, 17-H), 0.72 (3H, s, 18-H), 1.05 (3H, s, 19-H), 1.43 (1H, m, 20-H), 0.88 (3H, d, *J* = 6.1 Hz, 21-H), 1.13, 1.56 (2H, m, 22-CH_2_), 1.90, 2.04 (2H, m, 23-CH_2_), 5.58 (1H, m, 24-H), 1.68 (3H, s, 26-H), 1.61 (3H, s, 27-H), 0.99 (3H, s, 28-H), 0.88 (3H, s, 29-H), 0.97 (3H, s, 30-H). ^13^C-NMR: 34.6 (C-1), 27.4 (C-2), 78.0 (C-3), 38.8 (C-4), 48.2 (C-5), 35.8 (C-6), 198.3 (C-7), 138.9 (C-8), 165.4 (C-9), 39.3 (C-10), 23.7 (C-11), 29.9 (C-12), 44.6 (C-13), 47.7 (C-14), 31.4 (C-15), 28.7 (C-16), 48.2 (C-17), 15.7 (C-18), 18.6 (C-19), 35.6 (C-20), 18.8 (C-21), 35.5 (C-22), 24.7 (C-23), 125.1 (C-24), 139.4 (C-25), 25.7 (C-26), 17.7 (C-27), 27.3 (C-28), 15.1 (C-29), 24.4 (C-30).

*(24R)-Eupha-8,25-diene-3β,24-diol* (**5**), Formula: C_30_H_50_O_2_; colorless oil; [α]_D_^20^ +5.1° (c 0.30, MeOH); ESI-MS *m/z*: 465 [M+Na]^+^; the ^1^H-NMR and ^13^C-NMR data in accordance with the literature [24]. ^1^H-NMR: 4.92, 4.83 (2H, br s, 26-CH_2_), 4.01 (1H, t, *J = *6.1 Hz, 24-H), 3.23 (1H, dd, *J* = 4.5, 11.7 Hz, 3-H), 1.72 (3H, br s, 27-H), 0.99, 0.94, 0.86 (each 3H, s, CH_3_), 0.85 (3H, d, *J* = 6.2 Hz, 21-H), 0.79, 0.75 (each 3H, s, CH_3_); ^13^C-NMR: 35.3 (C-1), 27.9 (C-2), 79.0 (C-3), 38.9 (C-4), 51.0 (C-5), 18.9 (C-6), 27.7 (C-7), 134.0 (C-8), 133.5 (C-9), 37.3 (C-10), 21.5 (C-11), 30.9 (C-12), 44.1 (C-13), 50.0 (C-14), 31.1 (C-15), 28.0 (C-16), 49.7 (C-17), 15.7 (C-18), 20.1 (C-19), 36.0 (C-20), 19.0 (C-21), 37.3 (C-22), 31.6 (C-23), 76.6 (C-24), 147.6 (C-25), 111.1 (C-26), 17.4 (C-27), 28.0 (C-28), 15.5 (C-29), 24.5 (C-30).

*(20R,23E)-Eupha-8,23-diene-3β,25-diol* (**6**), Formula: C_30_H_50_O_2_; colorless needles; m.p. 132–133 °C; [α]_D_^20^ +17.0° (c 0.45, CHCl_3_); ESI-MS *m/z*: 465 [M+Na]^+^; the ^1^H-NMR and ^13^C-NMR data in accordance with the literature [24]. ^1^H-NMR: 5.58 (2H, br s, H-23 and H-24), 3.23 (1H, dd, *J =* 4.5, 11.7 Hz, 3α-H), 2.34 (1H, br d, *J = *12.7 Hz, 5-H), 1.31 (6H, s, 26-H and 27-H), 1.00, 0.95, 0.88 (each 3H, s, 30-H, 28-H, and 29-H), 0.82 (3H, d, *J = *6.0 Hz, 21-H), 0.80, 0.78 (each 3H, s, 19-H and 18-H). ^13^C-NMR: 35.3 (C-1), 27.9 (C-2), 79.0 (C-3), 38.9 (C-4), 51.0 (C-5), 18.9 (C-6), 27.7 (C-7), 134.0 (C-8), 133.5 (C-9), 37.3 (C-10), 21.5 (C-11), 31.0 (C-12), 44.2 (C-13), 50.0 (C-14), 29.8 (C-15), 27.9 (C-16), 49.6 (C-17), 15.8 (C-18), 20.2 (C-19), 35.7 (C-20), 19.1 (C-21), 37.3 (C-22), 125.7 (C-23), 139.3 (C-24), 70.8 (C-25), 29.9 (C-26), 29.9 (C-27), 28.1 (C-28), 15.5 (C-29), 24.5 (C-30).

### 3.4. Computational Methods

AutoDock Vina 1.1.2 [25] was used for all dockings in this study. The 1.80 Å X-ray structure human 11β-HSD1 complexed with inhibitor (PDB code: 3TFQ) was chosen as receptor in docking simulations. The Ligand and receptor were prepared with MGLTools 1.5.2. In general, the docking parameters for Vina were kept to their default values. The center of docking-grid is located on the centric of the corresponding ligand. The top 10 poses of each compound were reserved for the binding mode analysis. DiscoveryStudio 2.5.5 was used to build up the sequence alignment between human 11β-HSD1 and HSD2. The final structures were analyzed using PyMOL [26].

### 3.5. Biological Testing of Compounds

Glucocorticoid hormones play important roles in many biological and physiological processes, including regulation of energy metabolism; inflammatory, immune and stress responses; and cardiovascular homeostasis. The action of glucocorticoid on target tissue is not inevitable dependent on the circulating levels, but is regulated in a tissue-specific manner by the 11β-hydroxysteroid dehydrogenase enzymes (11β-HSD1 and 11β-HSD2), which catalyze the interconversion of active 11-hydroxyglucocorticoids (cortisol in human and corticosterone in rodent) and their respective inert 11-keto forms (cortisone in human and 11-dehyfrocorticosterone in rodent) [27]. 11β-HSD1 is highly expressed in liver, gonad, adipose tissue and brain, where it acts as a reductase regenerating the active glucocorticoids from its inactive forms, thus amplifies local glucocorticoid action [28]. 11β-HSD2 is predominantly expressed in aldosterone target cells such as kidney and colon, where it catalyses the inactivation of glucocorticoids, thereby preventing excessive activation of the mineralocorticoid receptor and sequelae including sodium retention, hypokalemia, and hypertension. We tested the inhibitory effect of compounds on both human and mouse 11β-HSD1 and 11β-HSD2. All tests were done duplicate with glycyrrhizininc acid (human and mouse 11β-HSD1, human 11β-HSD2) and carbenoxolone (mouse 11β-HSD2) as positive control. IC_50_ (X ° S.D., n = 2) values were calculated by using Prism Version 4 (GraphPad Software, San Diego, CA, USA.).

Inhibition of compounds on human or mouse 11β-HSD1 and 11β-HSD2 enzymatic activities were determined by the scintillation proxitimity assay (SPA) using microsomes containing 11β-HSD1 or 11β-HSD2 according to the previous studies [29]. Briefly the full-length cDNAs of human or marine 11β-HSD1 and 11β-HSD2 were isolated from the cDNA libraries provided by NIH Mammalian Gene Collection and cloned into pcDNA3 expression vector. HEK-293 cells were transfected with the pcDNA3-dericed expression plasmid and selected by cultivation in the presence of 700 μg/mL of G418. The microsomal fraction overexpressing 11β-HSD1 or 11β-HSD2 was prepared from the HEK-293 cells stable transfected with either 11β-HSD1 or 11β-HSD2 and used as the enzyme source for SPA. Microsomes containing human or mouse 11β-HSD1 was incubated with NADPH and [3H]cortisone, then the product, [3H]cortisol was specifically captured by a monoclonal antibody coupled to protein A-coated SPA beads. The 11β-HSD2 screening was performed by incubating 11β-HSD2 microsomes with [3H]cortisol and NAD^+^ and monitoring substrate disappearance. IC_50_ (X ± S.D., n = 2) values were calculated by using Prism Version 4 (GraphPad Software, San Diego, CA, USA.) with glycyrrhizininc acid and carbenoxolone as positive control.

## 4. Conclusions

We have described the isolation and characterization of six euphane-type triterpenoids from the plant *Euphorbia kansui*. All compounds were fully characterized using MS, ^1^H- and ^13^C-NMR spectroscopic techniques. In bioassay studies, all the compounds exhibited strong inhibitory activity against human 11β-HSD1 (11β-hydroxysteroid dehydrogenase type 1). The molecular weight of these six compounds is less than 500 and the number of hydrogen bond donors and acceptors is less than 5. In conclusion, the obtained results indicate that *E. kansui* exhibits potent inhibitory activities against human 11β-HSD1 which might be useful for therapeutic purposes to prevent type 2 diabetes, and could be used as a potential antidiabetic agent for treatment of diabetes. 

## Supplementary Materials

Supplementary materials can be accessed at: http://www.mdpi.com/1420-3049/17/10/11826/s1.
